# Fast and Long‐Term Super‐Resolution Imaging of Endoplasmic Reticulum Nano‐structural Dynamics in Living Cells Using a Neural Network

**DOI:** 10.1002/smsc.202400385

**Published:** 2024-11-08

**Authors:** Johanna V. Rahm, Ashwin Balakrishnan, Maren Wehrheim, Alexandra Kaminer, Marius Glogger, Laurell F. Kessler, Matthias Kaschube, Hans‐Dieter Barth, Mike Heilemann

**Affiliations:** ^1^ Institute of Physical and Theoretical Chemistry Goethe University Frankfurt Max‐von‐Laue‐Str. 7 60438 Frankfurt Germany; ^2^ Department of Computer Science and Mathematics Goethe University Frankfurt 60054 Frankfurt Germany; ^3^ Frankfurt Institute for Advanced Studies (FIAS) 60438 Frankfurt Germany; ^4^ International Max Planck Research School (IMPRS) on Cellular Biophysics Max Planck Institute of Biophysics Max‐von‐Laue‐Str. 3 60438 Frankfurt Germany; ^5^ Optical Imaging Competence Centre Friedrich‐Alexander‐Universität Erlangen‐Nürnberg Cauerstraße 3 91058 Erlangen Germany

**Keywords:** autophagy, denoising, image restoration, live‐cell microscopy, neural networks, super‐resolution microscopy

## Abstract

Stimulated emission depletion (STED) microscopy is a super‐resolution technique that surpasses the diffraction limit and has contributed to the study of dynamic processes in living cells. However, high laser intensities induce fluorophore photobleaching and sample phototoxicity, limiting the number of fluorescence images obtainable from a living cell. Herein, these challenges are addressed by using ultra‐low irradiation intensities and a neural network for image restoration, enabling extensive imaging of single living cells. The endoplasmic reticulum (ER) is chosen as the target structure due to its dynamic nature over short and long timescales. The reduced irradiation intensity combined with denoising permits continuous ER dynamics observation in living cells for up to 7 h with a temporal resolution of seconds. This allows for quantitative analysis of ER structural features over short (seconds) and long (hours) timescales within the same cell, and enabled fast 3D live‐cell STED microscopy. Overall, the combination of ultralow irradiation with image restoration enables comprehensive analysis of organelle dynamics over extended periods in living cells.

## Graphical abstract

Photobleaching and phototoxicity limit the observation time of living cells with super‐resolution fluorescence microscopy. Herein, the authors use low‐illumination microscopy and neural network assisted denoising to achieve fast and long‐term stimulated emission depletion microscopy in living cells. Imaging of the endoplasmic reticulum in live cells at sub‐100 nm spatial resolution and and ≈4 s time resolution over 7 h is demonstrated.

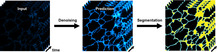

## 1 Introduction

Super‐resolution microscopy (SRM) has enabled visualizing cellular structures beyond the diffraction limit and has been established as an important tool in cell biology research.^[^ ^1^ ^]^ However, static structures in a cell only reveal part of the picture as biomolecules and cellular structures in a cell are highly dynamic. Understanding these dynamics is therefore crucial for attaining a comprehensive mechanistic picture. Stimulated emission depletion (STED) microscopy is one SRM method that can be employed in living cells and can theoretically achieve unlimited spatial resolution.^[^ ^2^ ^]^ This resolution is achieved by employing high excitation laser powers (≈kW cm^−2^) and very high depletion laser powers (up to ≈MW cm^−2^), which leads to photobleaching and phototoxicity.^[^ ^3^ ^]^ In addition, the acquired data could be artifact‐ridden as a high laser illumination might induce cellular processes that influence biomolecules in a cell. Various strategies have been introduced to mitigate both photobleaching and phototoxicity. Two simple methods involve engineering highly photostable organic fluorophores^[^ ^4^ ^]^ or using transient, weak‐affinity, and exchangeable labels or fluorophores.^[^ ^5^, ^6^, ^7^ ^]^ In the case of photostable fluorophores, photobleaching can only be relieved in the short‐term, whereas in the case of transient labels photobleaching can be circumvented relatively longer due to the exchange of fluorophores being sampled. In both cases though, laser powers remain more or less the same and hence, phototoxicity is still a hurdle in the long‐term (approximately several minutes). Techniques have been developed that involve minimal sampling which is either controlled by the presence of signal^[^ ^8^, ^9^ ^]^ or by the initiation of an event^[^ ^10^ ^]^ thereby reducing the overall illumination laser intensity in a cell. However, over the long‐term of several hours, phototoxicity still remains. One method that might be promising on this aspect is content‐restoration (denoising) of images recorded with low laser intensities (resulting in noisy images), which can be achieved with neural networks.^[^ ^11^, ^12^, ^13^, ^14^ ^]^ Although the maximum time acquisition shown in the case of STED has been limited to several minutes,^[^ ^14^ ^]^ the technique should enable long‐term imaging over several hours due to the decrease in laser intensity incident on the sample.

An additional concern, especially in live cells, is the speed of imaging which determines the accessible temporal dynamics that can be observed in live cells. Focusing on organelle dynamics, the optimum imaging condition would be the one that gives the best possible resolution without any movement artifacts. Depending on the organelle, this could mean that STED nanoscopy yields only sub‐par resolution with decreased signal. One such organelle that is highly dynamic in an eukaryotic cell is the endoplasmic reticulum (ER). The ER is the largest organelle in an eukaryotic cell and encompasses different functions ranging from protein folding, translocation and secretion to lipid synthesis, and Ca^2+^ signaling.^[^ ^15^ ^]^ Its structure varies from tubes, sheets, and matrices^[^ ^16^, ^17^ ^]^ and has been shown to restructure itself in the order of seconds.^[^ ^18^ ^]^ To capture the dynamics of the ER, very fast imaging would be required, which in turn means collecting fewer photons from a sample. The integration of a neural network that restores the image content through denoising would mean an increase in the speed of imaging, making it possible to follow organelles like the ER with STED nanoscopy with very minimal to no movement artifacts.

Over the last few years, there have been tremendous improvements in integrating various imaging strategies and their outputs with neural networks for augmented microscopy, image‐restoration, generation, segmentation, etc.^[^ ^11^, ^19^, ^20^, ^21^, ^22^, ^23^ ^]^ Two prominent neural networks that have been established for image restoration through denoising and feature extraction via segmentation are UNet‐RCAN^[^ ^14^ ^]^ and ERnet,^[^ ^24^ ^]^ respectively. UNet‐RCAN employs a two‐step architecture for prediction where a U‐Net^[^ ^25^ ^]^ predicts the broad contextual information, while a residual channel attention network (RCAN)^[^ ^26^ ^]^ focuses on the fine details of the image. UNet‐RCAN has been shown to outperform commonly used supervised neural networks such as pix2pix^[^ ^12^ ^]^ and CARE^[^ ^11^ ^]^ in the task of denoising STED images and has been used to reconstruct noisy STED images of fixed and live‐cells in both single‐plane and volumetric imaging.^[^ ^14^ ^]^ ERnet, on the contrary, employs a vision transformer‐based architecture to classify image pixels as tubes, sheets, sheet‐based tubes (SBTs) and background widefield, confocal, and structured illumination microscopy (SIM) images of the ER.^[^ ^24^ ^]^

Here, we demonstrate long‐term live‐cell STED imaging of the ER using UNet‐RCAN for low‐intensity denoising and ERnet for segmentation and feature extraction of the ER in the predicted image and establish a robust pipeline for studying live‐cell ER dynamics. For this, we trained UNet‐RCAN networks using ground truth and low‐intensity STED images of live‐cell ER. This network, once trained, exhibits the capacity to generate super‐resolved images based on their low‐intensity counterpart. We report a mean restored resolution of 89 nm in the predicted live‐cell images. Whereas live‐cell organelle imaging using STED has been limited in mitochondria to 12 min with fluorophores like PKMO^[^ ^27^ ^]^ or 12.5 h (with a snapshot acquisition of one frame every 3.4 min) with MAO‐N_3_^[^ ^28^ ^]^ and in ER to ≈20 min with a ceramide lipid Cer‐HMSIR,^[^ ^29^ ^]^ we extend this further by acquiring dynamics of ER in resting cells for up to 7 h in 2D (time resolution: 4.6 s) and up to 17 min in 3D (time resolution: 10.2 s).

To conclude, we show that by combining neural network‐based denoising to low‐intensity STED nanoscopy ER dynamics can be observed over the span of several hours under super‐resolution and this data can be segmented by tools like ERnet. In addition, our work encompasses the need for incorporating deep learning‐based restoration and segmentation strategies to study long‐term live‐cell organelle dynamics beyond the diffraction limit.

## 2 Results

### 2.1 Denoising Pipeline for Live Cell ER STED Images

**Figure 1:**
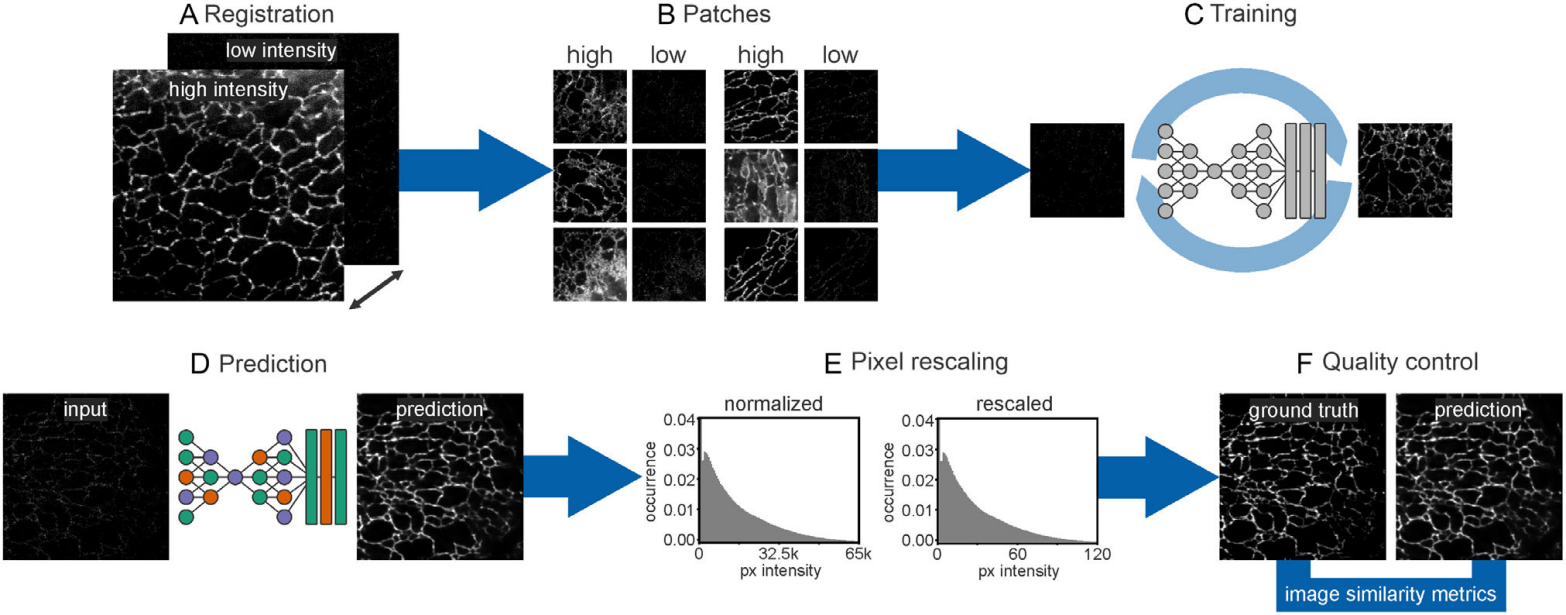
STED denoising workflow. A) Ground truth and the corresponding noisy images are aligned in the xy plane, B) from which small patches of high‐ and low‐intensity pairs are generated. C) A model is generated by training a UNet‐RCAN network, wherein the patches from low‐intensity images function as input and the high‐intensity patches serve as the training target and ground truth. An exploration of various hyperparameters is undertaken to identify a suitable model. D) The trained model is employed for prediction. E) The resulting outputs are rescaled to align with the pixel distribution of the ground truth. F) Quality‐control metrics are then computed on a designated test dataset to probe the model performance.

We introduce long‐term STED microscopy in live cells using low‐intensity image recording to minimize photobleaching and phototoxicity. For content restoration through denoising of low‐intensity images, the UNet‐RCAN network was chosen, as it preserves most of the resolution of the ground truth STED images compared to other methods.^[^ ^14^ ^]^ UNet‐RCAN is a supervised neural network, thereby requiring matching pairs of low‐intensity (noisy) and high‐intensity (ground truth) images (**Figure** **1**A). The noisy and ground truth image pairs were acquired sequentially with the acquisition switching between lines (line‐sequential acquisition) of the noisy and ground truth image to mitigate any spatial offset between the image pairs, which would dampen the quality of predictions. The rapid acquisition of noisy images at exceptionally high speeds (≈300–600 ms frame^−1^) minimizes discernible spatial shifts, while any residual offsets were drift corrected using the NanoJ's registration method.^[^ ^30^ ^]^ The acquired noisy and ground truth image pairs were then cropped into smaller patches (128 × 128, 256 × 256, or 304 × 304 pixels) and served as training data for the network (Figure 1B). This has the advantage of generating numerous training instances with only a few measurements. Furthermore, processing the entire recorded image is not possible due to limited RAM size on the GPU. To enhance the network's resilience to potential variations in samples across measurement days, inputs from at least three measurement days were used for training.

Initial attempts to train the UNet‐RCAN model using default parameters proved unsuccessful. The model did not converge and a large loss was observed, indicating gradient explosion and rendering the model not useful. Despite adjustments of hyperparameters (patch size, learning rate, kernel init., etc.), this issue persisted, necessitating the introduction of additional hyperparameters which were included in the UNet‐RCAN code base. An exploration of more than a hundred parameter combinations ensured the identification of a model that converges (Figure S1, Supporting Information) and performs adequately (Figure 1C).

Model performance was probed with a test dataset, which was acquired on a separate measurement day (Figure 1D). The generated predictions have normalized pixel intensities, which were rescaled to match the pixel distribution of the ground truth data (Figure 1E). The rescaling procedure exclusively leveraged information from the training data, preventing any influence from the test dataset. Finally, quality‐control metrics were computed on the rescaled predictions and ground truth data, facilitating the identification of an optimal model within the screened parameter space (Figure 1F).

Numerous models were trained with different hyperparameter combinations, allowing for the investigation of their importance regarding model quality with a random forest approach (Figure S2, Supporting Information). The two most important hyperparameters for model training are an appropriate learning rate and the size of the input. A patch size of 304 × 304 pixels (the size must be divisible by 8 due to the three down‐sampling layers in the U‐Net) was the optimal choice for this dataset, while larger patch sizes were limited by memory restrictions. Given the pivotal role of this parameter, we advocate for its optimization in conjunction with considerations of image content size, filter size, and downsampling extent. The importance of different parameter values within a parameter group is listed in Table S1 and S2, Supporting Information. This gives an overall indication of appropriate value choices per hyperparameter. However, the best performing models across the hyperparameter search result in slightly different combinations (Table S3, Supporting Information).

### 2.2 Evaluation of Predictions through a Comprehensive Assessment of Image Quality

**Figure 2:**
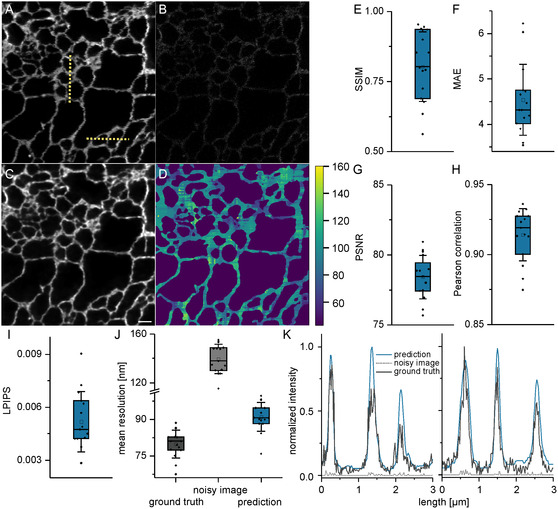
STED denoising of ER in live cells. A) Ground truth STED image of the ER of a U2‐OS cell. B) Noisy STED image of the ER of a U2‐OS cell acquired parallelly as a line acquisition during A. C) Predicted image of B. using the UNet‐RCAN network. D) rFRC resolution map corresponding to the predicted image, the color code represents the mean resolution in nm. The predicted image quality was probed using different control metrics such as E) SSIM; F) MAE; G) Pearson correlation; H) PSNR and I) LPIPS. J) Mean resolution of ground truth and predicted image calculated using rFRC. K) Intensity line profiles (along the yellow dotted lines in (A)) of ground truth (dark gray), noisy (dotted gray) and predicted image (blue), the intensities were normalized together as a single group. Scale bar: 1 μm, *N* = 15.

Since the focus of our study was on ER dynamics, we used U2‐OS cells stably expressing calreticulin‐KDEL as an ER marker conjugated to a HaloTag^[^ ^31^ ^]^ which was labeled with covalent HaloTag ligand bound to the fluorophore SiR as a fluorescent system. The live cells were in their normal resting state, which meant that there was minimal perturbation on the ER morphology. The final trained model on the resting cells is termed the resting model. **Figure** **2**A,B shows ground truth and noisy STED images respectively, of a part of the ER (12 × 12 μm^2^) in a representative cell. The resulting prediction using the resting model recovers the structural details in the ground truth image (Figure 2C).

A rolling Fourier ring correlation (rFRC) map^[^ ^32^ ^]^ of the predicted image displays how the resolution varies in different regions of the image giving a mean resolution of 89 nm and a minimum resolution of 42 nm (Figure 2D). In context, the corresponding ground truth image has a mean resolution of 82 nm. All predicted images were further scrutinized using different quality‐control (QC) metrics ranging from structural similarity index (SSIM),^[^ ^33^ ^]^ mean absolute error (MAE), peak signal‐to‐noise ratio (PSNR), and Pearson correlation to learned perceptual image patch similarity (LPIPS)^[^ ^34^ ^]^ (Figure 2E–I). MAE focuses on the pixel‐wise absolute difference between two images. PSNR evaluates the quality of a reconstructed signal, by comparing it to the original, uncompressed signal. The Pearson correlation evaluates the relationship of pixel intensities. Contrary to these metrics, SSIM and LPIPS consider spatial pixel dependencies. SSIM evaluates the similarity of the images in the aspects of structure, contrast, and luminescence. In LPIPS, feature representations extracted from pre‐trained neural networks are compared between prediction and ground truth images. This combination of different metrics, along with the spatial resolution, is a robust toolbox for comprehensive image quality assessment. All QC metrics show that the predictions recover the structural details of the ground truth image. In addition, the predictions also recover the resolution although not fully compared to the ground truth images (Figure 2J). Intensity profiles across the image also show that the predictions retain the underlying details of a ground truth image (Figure 2K).

### 2.3 Effects of Morphological Changes on Model Robustness

Subsequently, we wanted to explore how the quality of the network prediction is affected if the morphology of the ER undergoes structural changes. For this purpose, cells were treated with Torin1 and Bafilomycin to induce ER‐phagy.^[^ ^35^ ^]^ Noisy ground truth image pairs were acquired from autophagy‐induced cells for training a unique model for autophagy‐induced cells. Autophagy induction is a time‐dependent process where the ER of a cell changes continuously^[^ ^36^, ^37^ ^]^ since induction, therefore we went with a prediction model where the training data was partly from autophagy‐induced cells and partly from resting cells to account for diverse morphologies. This model was termed mixed model. The mixed model performed well with the test data from autophagy‐induced cells (Figure S3, Supporting Information) and the resting model with the resting cell test data (Figure 2). This performance extended in terms of structure recovery (Figure S3A–C, Supporting Information), resolution (Figure S3D, Supporting Information), intensity fluctuations (Figure S3E, Supporting Information) and QC metrics (Figure S4, Supporting Information).

To test the robustness of both the resting and the mixed model we predicted test input data from resting cells with the mixed model and autophagy‐induced cells with the resting model and evaluated their performance with QC metrics and resolution. In terms of QC metrics, the mixed model and resting model performed equally well for data of autophagy‐induced cells (Figure S4, Supporting Information). The model solely trained on resting cells performed slightly better on the resting cell test dataset (Figure S5, Supporting Information). Overall, the structural features were restored in all predictions (Figure S6, Supporting Information). Interestingly, movement artifacts that partly appear in the ground truth test data due to the fast organelle dynamics are averaged out in the predicted images. This shows that the data from both conditions was within the generalization capability of the resting model.

### 2.4 Strategies to Discern Structural Information from Hallucinations

To ensure the quality of predictions, it is crucial that the low‐intensity input still contains some signal that has not completely bleached away. In the absence of signal, hallucinations occur (Figure S7A,B, Supporting Information). To distinguish hallucinated predictions from those stemming from structure, the similarity of adjacent frames in a movie can be probed. Moving organelles exhibit a degree of similarity between adjacent frames, resulting in higher structural similarity when compared to adjacent predictions derived from empty field of views (FOVs) (Figure S7C, Supporting Information). By tracking the similarity over time the authenticity of predictions can be validated. Areas of uncertain predictions might not only arise temporally but also spatially; for instance, when a minute signal was collected from out‐of‐focus planes, which is not sufficient for a robust prediction (Figure S7D,E, Supporting Information). By thresholding the pixel intensities of the low‐intensity image, the pixels can be roughly grouped into background, uncertain, and signal pixels. This grouping gears the attention to areas with potential artifacts.

### 2.5 Denoising and Segmentation to Probe Fast and Long‐Term ER Dynamics

ER as an organelle exhibits fast dynamics visualized as rearrangement in structure and has been shown to happen in the order of seconds.^[^ ^18^ ^]^ In addition, the morphology of the ER is also profoundly responsive to conditions that induce stress and is in the order of hours to days. Taken together, it is important to observe ER dynamics with a time resolution of seconds over the span of hours. Therefore, as a first step into this direction, we extended our UNet‐RCAN implementation from super‐resolved planar images of the ER to study the long‐term temporal progression of ER dynamics in resting cells with a time resolution of seconds.

**Figure 3:**
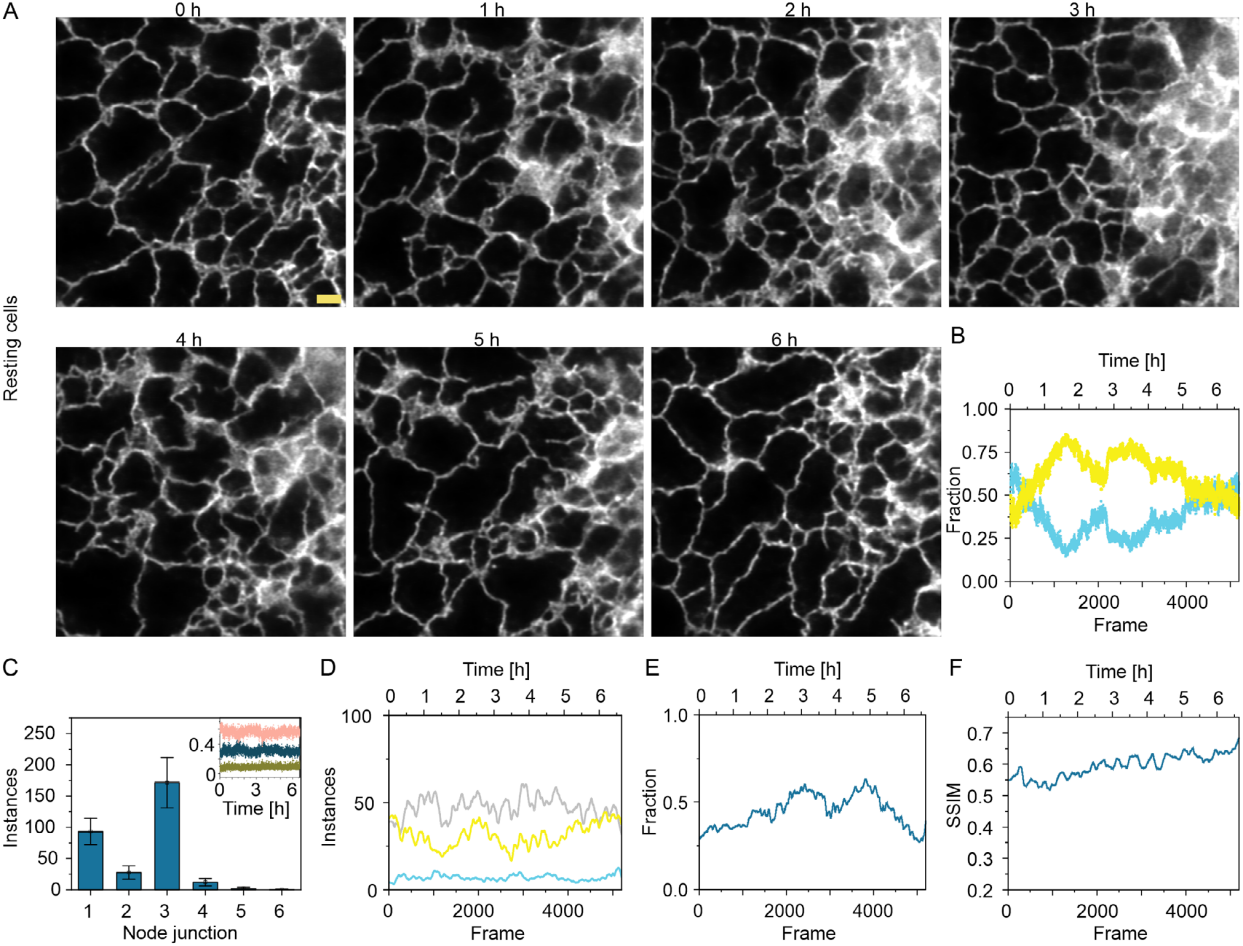
Long term denoising and segmentation of fast ER dynamics. A) Predictions of low‐intensity STED images of ER (calreticulin‐KDEL) at different time points, acquired on resting cells and predicted using the resting model. B) Fraction of tubes (cyan) and sheets (yellow) calculated using ERnet segmentation on (A). C) Amount of different tubular junctions calculated using ERnet segmentation averaged over all frames. (C), Inlay Fraction of one‐ (green), two‐ (blue), and three‐ (pink) way tubular junctions over the whole measurement time of (A). D) Instances of sheets (yellow), tubes (cyan) and gaps (light gray) calculated from (A). E) Fraction of foreground pixels in each frame in (A). F) SSIM scores for adjacent frames calculated for all frames in (A). *N* = 3. Scale bar: 1 μm.

We acquired low‐intensity STED images of the ER in normal resting cells for over 7 h with a temporal resolution of 4.6 s and denoised it using the UNet‐RCAN model trained on resting cells (**Figure** **3**A, Video S1, Supporting Information). We acquired this data for three different samples seeded over three different days to account for heterogeneity. ER rearrangements are variable in each cell even in their normal resting state (Figure 3A, S8x, Video S1–S3, Supporting Information).

We next aimed to quantify ER rearrangements over the full measurement time in more detail. As a start, we employed a neural network‐based segmentation tool, termed ERnet,^[^ ^24^ ^]^ on STED nanoscopy datasets. ERnet takes planar images as input and segments them into tubes, sheets, and sheet‐based tubes (SBTs). The segmented SBTs resembled part of sheets rather than tubular structures, probably due to the lack of real SBTs in the imaged area and the ERnet model being trained to detect SBTs. In our case, the small spots were merged with the segmented areas of sheets (Figure S9, Supporting Information). We note that we used an ERnet model trained on ER datasets acquired through SIM microscopy. Despite the differences in imaging modalities, the model demonstrated robust performance with our denoised STED data. ERnet segmentation shows that structural rearrangements are variable among cells in resting state. However, in all cases the amount of sheets and tubes fluctuates around a fraction of 0.5 (Figure 3B and S10Ai, ii, Supporting Information). In addition, we also see that the number of tubular junctions shows a majority at 3 (Figure 3C and S10Bi, ii, Supporting Information) as shown before^[^ ^24^ ^]^ and this feature stays constant over time (Figure 3C inlay, Figure S10Ci, ii, Supporting Information).

In addition to ERnet‐based segmentation, we analyzed structural rearrangements through various additional metrics such as instances of structures, the fraction of foreground pixels and SSIM score between adjacent frames. In the case of instances, the sheer number of sheets, tubes, and gaps in between tubes and sheets were calculated for each frame in the whole time series datasets. Figure 3D shows that the number of instances of tubes, sheets and the gaps (background enclosed by ER structure) between them stays more or less constant throughout the imaging time. In the other two cells recorded, we see more fluctuations in the case of gaps between tubes and sheets (Figure S10Di, ii, Supporting Information). The fraction of foreground pixels shows a trend similar to what we observe in the ERnet segmentation and is variable for the three different cells (Figure 3E and S10Ei, ii, Supporting Information). Here, we define foreground pixels as any non‐zero pixel in a binarized image of every frame. The SSIM scores reflect the frequency of changes happening in‐between frames. Figure 3F shows a slight increase in SSIM through the progression of the image acquisition. Data from other cells show heterogeneity with one staying constant throughout (Figure S10Fi, Supporting Information) and one showing a slight decrease (Figure S10Fii, Supporting Information) but overall SSIM values do not fluctuate drastically, reflecting that the frequency of structural rearrangement in resting cells is more or less constant.

### 2.6 Extending Denoising to Visualize Long‐Term ER Dynamics in 3D

The ER is a highly convoluted structure extending in 3D.^[^ ^16^ ^]^ Although 2D planar imaging over time gives insights into ER dynamics it does not completely reflect the underlying ground truth. Possible artifacts may arise from 2D projection of the dense 3D ER structure. Therefore, we extended the UNet‐RCAN model trained on 2D planar images (resting model) to denoise volumetric images from living cells over time.

We first tested the robustness of the resting model on singular volumetric datasets. The 3D stacks were limited to a thickness of 700 nm and only noisy images were acquired to keep spatial offset between frames to a minimum. In addition, a top‐hat point spread function (PSF) pattern was used for the depletion laser to gain axial resolution. Figure S11, Supporting Information, shows a predicted 3D stack of an exemplary cell visualized as *xy*, *yz*, and *xz* planes (Video S4, Supporting Information). Since only noisy images were acquired in the 3D stack, we measured additional single plane noisy and ground truth images (Figure S12, Supporting Information) with the same settings including a top‐hat depletion laser PSF as a control. We performed QC metrics on the control dataset (Figure S13, Supporting Information) to show that the resting model does indeed denoise images acquired with a top‐hat depletion laser PSF achieving a mean resolution of 89 nm (Figure S13F, Supporting Information).

**Figure 4:**
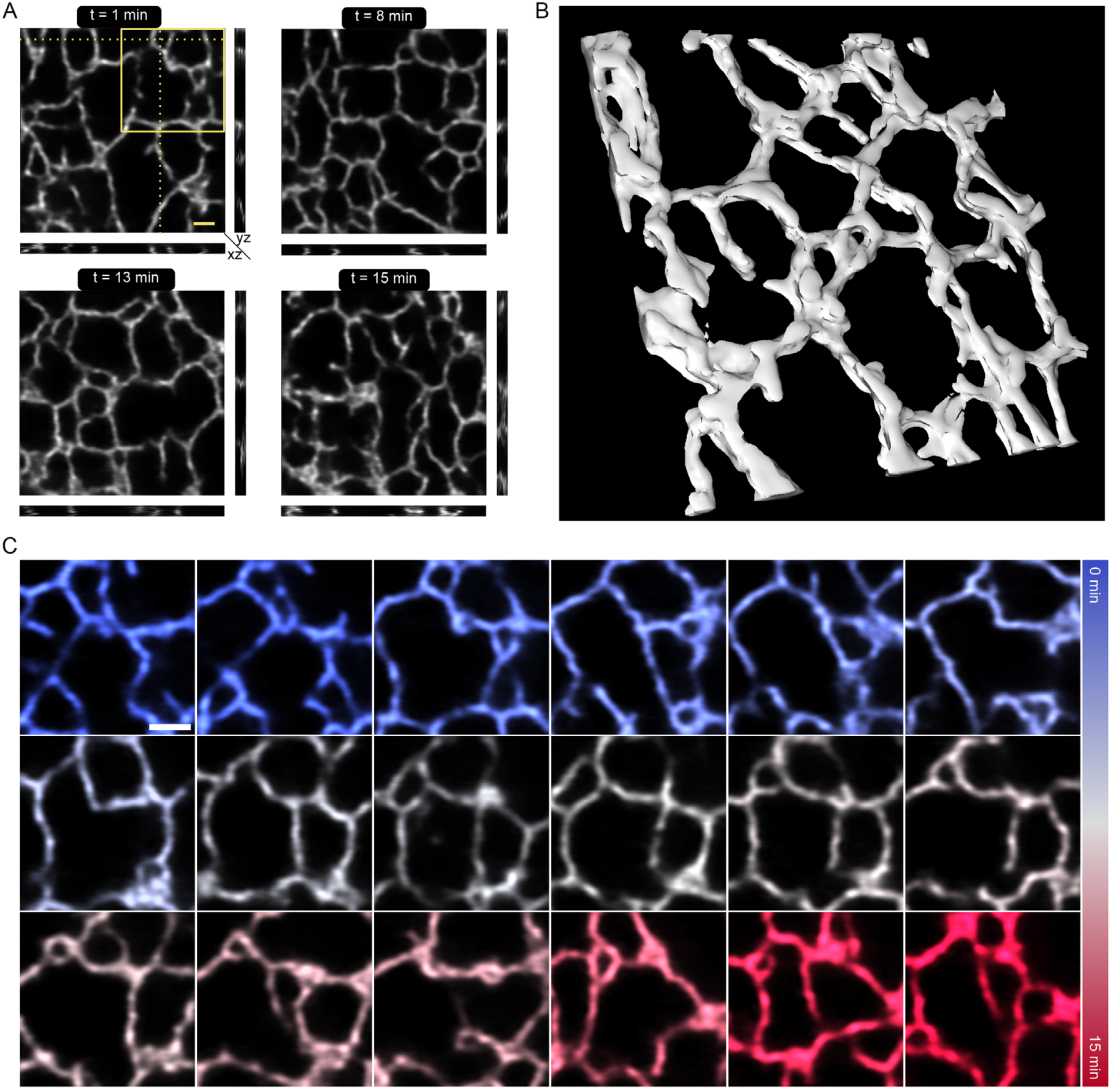
Denoising 3D stacks over time to visualize fast 3D ER dynamics. A) Orthogonal projections of predictions from *z*‐stacks of the ER acquired on live‐cells at different time points. B) Surface rendering of one of the *z*‐stacks shown in (A). C) Representative images from the inlay and the same z‐plane shown in (A) at the different time points throughout the acquisition. *N* = 4. Scale bars: 1 μm.

We then used the resting model to predict time series data of 3D stacks acquired over 17 min with a temporal resolution of 10.2 s. **Figure** **4**A shows orthogonal slices of an exemplary predicted data from 3D stacks over different points of time in the acquisition. The 3D surface rendering of the time series data clearly shows that considerable dynamics would be missed, for example, through projection, if it were not recorded in 3D mode (Figure 4B, Video S5, Supporting Information). Figure 4C highlights this point by visualizing certain structural rearrangements over time for one of the frames of the whole 3D stack. Similar data was acquired for different resting cells over different days (Video S6,S7, Supporting Information). Hence, we show that the resting model could be used on *z*‐stacks and extended to 3D time series over 17 min at super‐resolution.

To conclude, we implemented an UNet‐RCAN network to denoise STED images (planar‐ and volumetric imaging) of ER (calreticulin‐KDEL) in living cells and by reducing the laser intensity incident on the cells extend STED nanoscopy on live‐cells to over 7 h.

## 3 Discussion

Visualizing organelle dynamics in live‐cells with sub‐diffraction resolution and for extended time spans so far required either specialized microscopy equipment (lattice light sheet or grazing incidence SIM)^[^ ^18^, ^38^ ^]^ or intermittent sampling due to the prevalence of photobleaching and phototoxicity.^[^ ^28^ ^]^ An alternative approach is to restore image content using a neural network.^[^ ^11^, ^13^ ^]^ Previous work demonstrated that neural network assisted denoising can restore STED images and mitigate photobleaching and photodamage by reducing the pixel dwell time and hence light exposure. In addition, they showed that their UNet‐RCAN network outperformed pre‐available networks.^[^ ^14^ ^]^ The aim of our work was to expand live‐cell imaging to the time scale of hours and at the same time with high temporal resolution to visualize ER dynamics. By implementing an UNet‐RCAN network, we were able to measure live‐cells with ≈70 times lesser light dose per pixel while maintaining a sub‐100 nm resolution. We show that a model solely trained with resting cells is robustly predicting both data acquired from autophagy‐induced cells and normal resting cells respectively (Figure S4 and S5, Supporting Information). This shows that models generalize morphological changes to some extent, and acquiring training data from resting cells could be sufficient when working with multiple conditions. By leveraging the ≈70 times reduction in light dose and a faster acquisition speed of 600 ms per frame, we were able to measure low‐intensity STED images of ER (calreticulin‐KDEL) in living cells for over 7 h with minimal movement artifacts, mitigating photobleaching, and phototoxicity. To our knowledge, information on ER structural rearrangements with high spatial and temporal resolution over such long times was so far inaccessible.

We then predicted super‐resolution STED images from these low‐intensity images giving rise to time series data with sub‐100 nm spatial resolution. The predicted images had a ≈4 s time resolution for a time span of ≈7 h giving full structural information on the rearrangement of the ER. We accessed this by using ERnet‐based segmentation which is a neural network‐based semantic segmentation tool that has been shown to classify ER structures into tubes, sheets and SBTs especially from data acquired through SIM microscopy.^[^ ^24^ ^]^ In addition, through ERnet segmentation, Lu et al. have shown that three way tubular junctions are dominant in healthy ER and they observed that in 20% of the healthy cells that they analyzed exhibited ER with higher order tubular junctions.^[^ ^24^ ^]^ Both of these conditions are similar to what we observe in our time series data, where the three way tubular junctions dominate and higher order junctions are also evident (Figure 3C and S10B, Supporting Information). We also show that structural rearrangements could be analyzed through other metrics such as the foreground pixels and the SSIM index. Whereas the foreground pixel information is similar to that extracted from the ERnet, though not as detailed, the SSIM index gives information on the frequency of rearrangements occurring between frames. We see that for two of the three cells measured, the SSIM index stays constant throughout the measurement indicating that the structural rearrangements happening are not fluctuating from the mean. In the case of the outlier, we can see from Video S3, Supporting Information, that the cell itself moves and the amount of ER in the FOV is decreased which could lead to the decrease in the SSIM index. In addition, we also used the resting model for predicting volumetric data acquired from living cells. It can be seen from the control test data (Figure S13F, Supporting Information) that the mean resolution achieved is 89 nm. We extended this further by applying it to volumetric time series data for up to 17 min. We can see that certain structural rearrangements could not be comprehended if it were not for volumetric imaging (Figure 4C, Video S5–S7, Supporting Information). Volumetric imaging might prove useful especially when studying interorganelle dynamics. Taken together, the resting model we trained with the UNet‐RCAN network is robust enough to predict not only 2D planar images but also 3D volumetric images and 2D planar images from stress‐induced cells. This approach not only enables the identification and quantification of ER structural rearrangements but also how these are affected by cellular stress.

We chose the ER as the target organelle due to its dynamics being largely unknown. The morphology of the ER is profoundly responsive to conditions that induce stress within a cell. In this context, the ER plays an important role in stress‐induced autophagy, a cellular process, which involves the degradation and recycling of cellular components, defined as ER‐phagy. Only fixed cell studies have been performed on ER‐phagy samples under super‐resolution to the best of our knowledge.^[^ ^35^, ^39^ ^]^ Our pipeline that we developed here would serve in the future to visualize fast and long‐term ER dynamics under different stress‐induced conditions with different cell line backgrounds. Our work mainly focused on the ER which was recorded in a single fluorescence channel. We envision extending this approach to multiple spectral channels or add additional measurement parameters to study interorganelle dynamics or intraorganelle dynamics by selecting multiple targets of interest. UNet‐RCAN could be trained on live‐cell data from other organelles which can then be coupled to pre‐existing organelle‐specific segmentation tools^[^ ^40^, ^41^, ^42^ ^]^ thereby opening the possibility of one‐shot multi‐organellar dynamics and drawing a more holistic understanding of organelle dynamics and interactions. We also note that content restoration and denoising are areas of active research in the field of neural network assisted bioimage analysis, and we anticipate that alternative approaches can be integrated into this workflow.^[^ ^20^, ^43^, ^44^ ^]^ A point worth mentioning is that although we mitigated phototoxicity by using an ≈70 times reduced illumination light dose, the extent of this mitigation was not measured. Several studies have assessed the influence of phototoxicity on cells using different microscopy modalities.^[^ ^45^, ^46^, ^47^ ^]^ Kilian et al. in particular, observed the effects of STED illumination by monitoring Ca^2+^ levels in the cytoplasm as a stress indicator.^[^ ^45^ ^]^ The application of such an approach to the low‐intensity timeseries datasets acquired in this work could prove useful to understand to what degree phototoxicity was reduced.

In summary, our work demonstrates a neural network‐based workflow accessing the fast ER dynamics with long time observation at high resolution in planar and volumetric image acquisitions. This robust framework can be readily applied to other cellular structures, where fast dynamics with sub‐100 nm resolution are of interest.

## 4 Experimental Section

4.1

4.1.1

##### Cell Culture

Live‐cell samples were prepared from U2‐OS cells stably expressing Calreticulin‐KDEL‐HaloTag7 which has been used before in a previous work.^[^ ^31^ ^]^ The cell line was cultured in DMEM/F12 media supplemented with 10% fetal bovine serum (Corning, USA), 1% v/v penicillin‐streptomycin (Thermo Fisher Scientific, Germany) and 5% v/v Glutamax (Thermo Fisher Scientific, Germany). The cells were incubated at 37 °C and 5% CO_2_ and were passaged every 2–3 days or when they reached 80% confluency.

##### Sample Preparation for Live‐Cell Microscopy

U2‐OS cells stably transfected with calreticulin‐KDEL‐HaloTag7 were seeded onto fibronectin‐coated (Sigma‐Aldrich, Germany, 0.1% v/v fibronectin human plasma for 30 min) 8‐well chambered coverglass (Sarstedt, Germany) at an amount of 1 × 10^4^ cells well^−1^. The cells were incubated overnight at 37 °C and 5% CO_2_ and were induced the next day with 250–500 μg mL^−1^ of doxycycline (Sigma Aldrich, Germany) in culture media. Two days post‐induction, the cells were incubated for 15–20 min with 300 nm of HaloTag ligand conjugated to the fluorophore SiR diluted in culture media. The cells were then washed thrice with culture media with 5 min incubation at 37 °C with 5% CO_2_ between every wash. The cells were incubated in culture media at 37 °C with 5% CO_2_ till they were taken out for imaging. For imaging, the culture media was then exchanged for live‐cell media (reduced culture media with HEPES, Thermo Fisher Scientific, Germany) and the cells were imaged within the next 2 h unless explicitly mentioned.

##### Autophagy Induction

For autophagy induction, the media of the seeded cells were exchanged with live‐cell media containing final concentrations of 250 nm Torin1 (Bio‐techne, USA) and 250 nm BafA1 (Cayman Chemical Corporation, USA). The cells were imaged immediately to observe the progression of autophagy over time.

##### STED Microscopy

An Abberior expert line microscope (Abberior Instruments, Germany) was used for performing confocal and STED imaging. The setup had a Olympus IX83 body (Olympus Deutschland GmbH, Germany) where the imaging was done using a UPLXAPO 60x NA 1.42 oil immersion objective (Olympus Deutschland GmbH, Germany). Sample images were acquired by exciting with a 640 nm excitation laser (both confocal and STED imaging) and depleting (in the case of STED images) with a 775 nm laser which either had a donut PSF (for planar and long‐term imaging) or a top‐hat PSF (for volumetric imaging and planar control) and with a delay of 750 ps and fluorescence photons between 750 ps and 8.75 ns were detected between every laser pulse. Fluorescence was collected in the spectral range of 650–760 nm using an avalanche photo diode (APD). The power levels of the lasers used and the parameters used for imaging are summarized in **Table** **1**. Note that the power levels for the lasers were measured at the back focal plane. Training data was imaged in a 7.5 × 10 and 12 × 12 μm^2^ field of view (FOV); test data was imaged in a FOV of 12 × 12 μm^2^; 2D time series data was imaged in either 12 × 12 μm^2^ or 15 × 15 μm^2^ FOVs and 3D time series was imaged in a FOV of 10 × 10 μm^2^. All planar and volumetric timeseries datasets were acquired on the microscope with a caged live‐cell incubator (Okolab, Italy) which maintained the temperature at 37 °C and 5% CO_2_. The 8‐well chambered coverglass was kept covered with a lid during the measurement and did not exhibit evaporation of medium.

**Table 1:** Imaging parameters used for confocal and STED imaging.

Imaging modality and condition	Excitation laser power [μW]	Depletion laser power [mW]	Depletion laser PSF pattern	Line accumulation/integration	Pixel dwell time [μs]	Pinhole diameter (airy unit (AU))	Pixel size/Voxel size [nm]
Ground truth, single plane	56.3	166	Donut	6	0.5	0.81	20
Low intensity, single plane; timeseries	4.8	166	Donut	1	0.5	0.81	20
Low intensity, *z*‐stack	4.8	166	top‐hat	1	0.5	0.81	20
Ground truth, single plane	56.3	166	top‐hat	6	0.5	0.81	20
Low intensity, single‐plane	4.8	166	top‐hat	1	0.5	0.81	20
Low intensity, *z*‐stack, timeseries	4.8	166	top‐hat	1	0.5	0.81	35

##### Image Processing

High‐intensity and low‐intensity images were registered with NanoJ‐Core's registration method.^[^ ^30^ ^]^ For the resting condition, training images were acquired from three measurement days. For the mixed model, 26% of images were added from the treated condition. The same amount of data was used for training to allow comparison between the resting and mixed model performance. Images were cropped into patches of 128 × 128, 200 × 200, 256 × 256, and 304 × 304 pixels to determine which patch size is optimal. A training and validation split of 90–10% was made. Per condition, 15 test images of size 600 × 600 px^2^ were acquired at a different measurement day.

##### Network Training

UNet‐RCAN networks^[^ ^14^ ^]^ were trained on a NVIDIA RTX 3090 24 GB GPU. As the default values led to exploding gradients, we added some additional hyperparameter options to the published codebase, and the parameter space was explored to find a robustly working model. This included different loss functions (leaky ReLU, tanh), kernel initialization strategies (glorot uniform, lecun uniform, orthogonal), gradient clipping (0.1, 0.01, 0.001), and l2 regularization (0, 0.01, 0.001). Different learning rates were additionally tested during the search (0.001, 0.0001, 0.00001 for the resting and 0.001, 0.0001, 0.0005, 0.00005, 0.00001, 0.000001 for the mixed condition), along with different patch sizes (128 × 128, 200 × 200, 256 × 256, and 304 × 304 pixels). The batch size was chosen as the maximum possible number per patch size before an out‐of‐memory error occurred on the GPU (patch size:batch size, 128:36, 200:16, 256:10, 304:6).

A parameter space search was executed to identify possible parameter combinations for models that perform well on the test dataset. To diminish the search time, 1000 data instances were included and training was interrupted after 10 epochs or if the loss exceeded a value of 1000 during training. 147 models were evaluated for the resting condition and 393 models for the mixed condition. QC metrics were calculated on a test dataset after every model run to determine the model performance. The metrics SSIM,^[^ ^33^ ^]^ MAE, and the resolution (determined with decorrelation analysis^[^ ^48^ ^]^ in NanoPyx v0.2.2^[^ ^49^ ^]^) were each linearly transformed between 0 and 1 (0 means worst and 1 means best for all scores) across all model runs per condition. A “total score” was computed by summing the transformed scores and dividing them by three to assess performance across multiple metrics. The top five models, based on the highest total score, were trained until convergence on the full dataset with 1764 instances.

We have added information regarding model training and evaluation to the UNet‐RCAN public repository (https://github.com/vebrahimi1990/UNet_RCAN_Denoising/tree/master).

For the final calculation of quality‐control metrics, the pixel intensities of the predicted test images must be rescaled, as the model outputs normalized intensities. A scale and offset were calculated based on the pixel values of predicted training instances and their ground truth images; the test dataset is deliberately excluded from this process. A linear scaling operation was applied to the pixel values of the predicted images. Values above the 99th quantile and below the 1st quantile were clipped. The scale and offset values were determined per model and applied to the test data before calculating QC metrics. The final model was selected based on the highest total score, with parameters detailed in Table S3, Supporting Information.

##### Quality Control Metrics

The MAE was calculated using the mean absolute difference of pixel values. SSIM^[^ ^33^ ^]^ and PSNR were computed using the scikit‐image library v0.19.3.^[^ ^50^ ^]^ Pearson correlation was determined with SciPy v1.9.3.^[^ ^51^ ^]^ The learned perceptual image patch similarity (LPIPS)^[^ ^34^ ^]^ was ascertained with a VGG model. Resolution during model training was determined with decorrelation analysis^[^ ^48^ ^]^ in NanoPyx v0.2.2.^[^ ^49^ ^]^ Resolution for the test data was determined using rolling Fourier ring correlation v0.2.5^[^ ^32^ ^]^ with a 1/7‐threshold and a background intensity of 20.

To discern predictions stemming from FOVs with signal and FOVs containing only noise, the SSIM was calculated between adjacent predicted frames of the resting movie and a movie recorded of only dark noise of the detector (in an empty image plane without sample). To spatially group pixels into the categories background, uncertain, and signal, a sliding window of size 10 × 10 pixels was used to sum the pixel intensities within this window per pixel. Borders of the image were padded with reflected pixels to maintain the input size. A pixel was labeled as background, if the sum of the pixel intensities was equal or below 5, as uncertain if it was in between 6 and 25, and as signal if it was above 25. In the case where ground truth images were available, thresholds were determined based on the ground truth and low‐intensity images. When ground truth images were not available, thresholds were user determined based on the low‐intensity images. The window size and thresholds must be adapted per use case.

##### Feature Importance

The RandomForestRegressor from scikit‐learn v1.0.2^[^ ^52^ ^]^ was used to determine hyperparameter importance based on the 147 model runs for the resting and 393 model runs for the mixed condition. It ranks the hyperparameters based on their importance in achieving a high “total score”. Furthermore, suitabilities are assigned to the different parameter options within a parameter group.

##### Structural Analysis of the ER

Predictions of the long‐term planar timeseries datasets were segmented using ERnet^[^ ^24^ ^]^ to describe the underlying structural properties of the ER. The network architecture for ERnet has been described previously in literature.^[^ ^24^ ^]^ We performed a preprocessing step before segmentation with ERnet. A rolling ball background subtraction with a radius of 15 px was applied to the predicted images in Fiji.^[^ ^53^ ^]^ This prevents high background regions from being falsely classified as ER sheets. The background subtracted images were segmented using ERnet by utilizing a previously published model,^[^ ^24^ ^]^ 20220306_ER_4class_swinir_nch1.pth ERnet v2.0 that was pretrained with ER data acquired on a structured illumination microscope (SIM). The ERnet segmentation was performed on a Google Colab notebook provided by Lu et al.^[^ ^24^ ^]^ The segmentation of ERNet is based on a morphological analysis and classifies each structure within an image into tubules, sheets, and sheet‐based tubules (SBTs). Linear branched structures are classified as tubes, planar structures as sheets and sheets with tubular structures in between them as SBTs. As the latter was not visible in the predictions from the timeseries datasets and ERNet just segmented some minor spots as SBTs, these spots were merged into the sheet segmentation.

##### Post Processing Volumetric Data

The predictions from volumetric datasets (Video S4–S7, Supporting Information) were checked for hallucination artifacts as shown in Figure S7E, Supporting Information, and peripheral frames were cropped to avoid them. The cropped 3D stacks were rendered as surfaces using the “3D Viewer” in Fiji with “Display as” set to surface, “Resampling factor” set to 2 and “Threshold” set to 50.

##### Statistical Analysis

All datasets were probed for normality by applying Shapiro–Wilk tests (*α* = 0.05) and Mann–Whitney‐U tests were used to compare the metrics of ground truth and predicted images. Levels of signiﬁcance were defined as: *p* > 0.05 no signiﬁcant difference (n.s.), *p* < 0.05 signiﬁcant difference (*), *p* < 0.01 very signiﬁcant difference (**), *p* < 0.001 highly signiﬁcant difference (***). All tests were performed in Origin Pro 2018b.

## Conflict of Interest

The authors declare no conflict of interest.

## Author Contributions

**Mike Heilemann**, **Ashwin Balakrishnan**, and **Johanna V. Rahm** conceived the study. **Ashwin Balakrishnan**, **Alexandra Kaminer**, and **Laurell F. Kessler** measured the microscopy datasets. **Johanna V. Rahm** set up the and trained the neural network. **Hans‐Dieter Barth** setup the GPU server. **Johanna V. Rahm** and **Ashwin Balakrishnan** performed data analysis and wrote the original draft manuscript. **Ashwin Balakrishnan**, **Johanna V. Rahm**, **Maren Wehrheim**, **Marius Glogger**, **Hans‐Dieter Barth**, **Matthias Kaschube**, and **Mike Heilemann** reviewed and edited the final manuscript. Mike Heilemann acquired funding and oversaw the project. **Johanna V. Rahm** and **Ashwin Balakrishnan** contributed equally to this work.

## Supporting information

Supplementary Material

## Data Availability

The data that support the findings of this study are openly available in Zenodo at https://doi.org/10.5281/zenodo.13120792, reference number 13120792.
